# Novel CAD-like enzymes from *Escherichia coli* K-12 as additional tools in chemical production

**DOI:** 10.1007/s00253-012-4474-5

**Published:** 2012-10-24

**Authors:** André Pick, Broder Rühmann, Jochen Schmid, Volker Sieber

**Affiliations:** Wissenschaftszentrum Straubing, Lehrstuhl für Chemie Biogener Rohstoffe, Technische Universität München, Schulgasse 16, 94315 Straubing, Germany

**Keywords:** Alcohol dehydrogenase, *Escherichia coli*, Metabolic engineering, YqhD, YahK, YjgB, Bulk chemicals

## Abstract

In analyzing the reductive power of *Escherichia coli* K-12 for metabolic engineering approaches, we identified YahK and YjgB, two medium-chain dehydrogenases/reductases subgrouped to the cinnamyl alcohol dehydrogenase family, as being important. Identification was achieved using a stepwise purification protocol starting with crude extract. For exact characterization, the genes were cloned into pET28a vector and expressed with N-terminal His tag. Substrate specificity studies revealed that a large variety of aldehydes but no ketones are converted by both enzymes. YahK and and YjgB strongly preferred NADPH as cofactor. The structure of YjgB was modeled using YahK as template for a comparison of the active center giving a first insight to the different substrate preferences. The enzyme activity for YahK, YjgB, and YqhD was determined on the basis of the temperature. YahK showed a constant increase in activity until 60 °C, whereas YjgB was most active between 37 and 50 °C. YqhD achieved the highest activity at 50 °C. Comparing YjgB and Yahk referring to the catalytic efficiency, YjgB achieved for almost all substrates higher rates (butyraldehyde 221 s^−1^ mM^−1^, benzaldehyde 1,305 s^−1^ mM^−1^). Exceptions are the two substrates glyceraldehydes (no activity for YjgB) and isobutyraldehyde (YjgB 0.26 s^−1^ mM^−1^) which are more efficiently converted by YahK (glyceraldehyde 2.8 s^−1^ mM^−1^, isobutyraldehyde 14.6 s^−1^ mM^−1^). YahK and even more so YjgB are good candidates for the reduction of aldehydes in metabolic engineering approaches and could replace the currently used YqhD.

## Introduction

The production of fuels and chemicals from biomass and such “greening” the chemical industry is an important issue of today. Within this context, the microbial production of industrially relevant alcohols has been the subject of many studies in recent years. Especially methods of metabolic engineering and synthetic pathway design have been applied to enable different microorganisms to produce molecules like ethanol, *n*-butanol (Berezina et al. 2010), isobutanol (Liao et al. 2010), different propane diols (Emptage et al. 2003; Nakamura and Whited 2003; Berríos-Rivera et al. 2003) and butane diols (Yim et al. 2011; Carothers et al. 2009; Nielsen et al. 2010), as well as aromatic alcohols like furfuryl alcohol (Heer et al. 2009). The final step of all these syntheses is the reduction of an aldehyde to an alcohol. One enzyme stands out in its utilization for this step: the *Escherichia coli* alcohol dehydrogenase YqhD (Jarboe 2010; Tang et al. 2009; Liao et al. 2010). Accordingly, this enzyme was discovered within a project directed by DuPont for the production of 1,3-propanediol using an engineered *E*. *coli* strain (Emptage et al. 2003; Nakamura and Whited 2003). YqhD showed to be the better candidate for the reduction of 3-hydroxypropionaldehyde compared to the designated DhaT from *Klebsiella pneumoniae* (Wang et al. 2005; Skraly et al. 1998). In our case, investigations of the reductive power of *E*. *coli* revealed additional activities that can be important for the microbial production of alcohols and that are especially suited for the reduction of multifunctional aldehyde compounds with partially even higher activity than YqhD. Here, we report the identification and characterization of two such enzymes from *E*. *coli*, the zinc-dependent alcohol dehydrogenases YahK and YjgB.

## Materials and methods

### Reagents

Restriction enzymes, alkaline phosphatase, phusion^TM^ polymerase, and T4 ligase are from New England Biolabs (Frankfurt, Germany). Taq polymerase was obtained from Rapidozym (Berlin, Germany). Oligonucleotides were from biomers.net (Ulm, Germany). DNase was obtained from Serva (Heidelberg, Germany). All chemicals were of analytical grade or higher quality and purchased from Sigma-Aldrich, Merck, or Carl Roth. All columns used for protein purification were from GE Healthcare (Munich, Germany).

### Strains and plasmids

The following strains were used during this work: *E*. *coli* K-12 W3110, KeioCollection BW25113 (Baba et al. 2006), *E*. *coli* XL1 Blue, and *E*. *coli* BL21(DE3). For cloning of the genes *yjg*B (GenBank^TM^ U14003.1), *yah*K (GenBank^TM^ U00096.2), and *yqh*D (Genbank^TM^ GQ478251.1), genomic DNA of *E*. *coli* K12 W3110 was used as PCR template. For cloning of *yjg*B, the primers F-NdeI-*yjg*B-E.c.- CGACAGCATATGTCGATGATAAAAAGCTATGCCGC and R-XhoI-*yjg*B-E.c.- GACGATCTCGAGTCAAAAATCGGCTTTCAACACCACGC, for *yah*K the primers F-NheI-*yah*K-E.c- GACAGGCTAGCATGAAGATCAAAGCTGTTGGTGC and R-XhoI-*yah*K-E.c.-GACGATCTCGAGTCAGTCTGTTAGTGTGCGATTATCG, and for yqhD the primers F-NheI-*yqh*D-E.c.- GACAGGCTAGCATGGCGAACAACTTTAATCTGCACAC and R-XhoI-*yqh*D-E.c.-GACGACTCGAGTTAGCGGGCGGCTTCGTATATACGG were used. PCR products were digested with *Nde*I or *Nhe*I and *Xho*I and cloned into pET28a(+) (Novagen), cut with the same enzymes, creating the plasmids pET28a-NH-*yjg*B-E.c, pET28a-NH-*yah*K-E.c., and pET28a-NH-yqhD-E.c. Multiplication of the plasmids was performed by *E*. *coli* XL1 Blue (Stratagene) in Luria–Bertoni medium containing 30 μg/ml kanamycin. The *E*. *coli* strain BL21(DE3) (Novagen) was used for expression.

### Isolation of genomic DNA from *E*. *coli* K-12 W3110

The genomic DNA from *E*. *coli* K-12 W3110 was isolated from cells of an overnight culture using the protocol of Chen and Kuo (1993).

### Protein identification

All purification steps of alcohol dehydrogenase activities (ADHs) from *E*. *coli* W3110 were performed using an ÄKTA UPC-900 FPLC-system (GE Healthcare, Munich, Germany) at room temperature. All buffers were filtered with 0.2-μm regenerated cellulose membranes. Fractions were stored at −20 °C.

#### Crude extract

*E*. *coli* W3110 was cultivated in Luria–Bertoni (LB) medium at 150 rpm and 37 °C. The cells were harvested by centrifugation at 4,580×*g* for 25 min at 4 °C. Pellets were stored at −20 °C. Ten grams of frozen cells were dissolved in 40 ml of 20 mM Tris-HCl (pH 9), 20 μL of 2 M MgCl_2_ and DNase in a final concentration of 50 μg/ml. Cell lysate was prepared with a cell disruptor (IUL constant systems) at 1.33 kbar. Removal of cell debris was performed by centrifugation (4,580×*g*, 15 min, 4 °C). Purification of cell lysate was performed by denaturation for 15 min at 60 °C in a water bath. Finally, the suspension was centrifuged at 21,100×*g* for 15 min at 4 °C and filtrated via 0.45-μm cellulose acetate membranes.

#### Anion exchange chromatography

The cell lysate was applied to a HiTrap Sepharose Q XL 1 ml column equilibrated with 20 mM Tris-HCl buffer (pH 9.0, 1 ml/min). After washing with 20 bed volume of equilibration buffer, elution was executed with eluent B (20 mM Tris-HCl, 1 M NaCl and pH 9.0), starting with 10 % for 20 min, followed by a linear gradient up to 60 % within 90 min, and column wash with 100 % for 10 min. The eluted fraction was monitored at 280 nm and sampled (every 1.5 ml) into a 96-deep-well microtiter plate for further analysis.

#### Hydrophobic interaction chromatography

Different fractions from the first purification, with the desired activity, were pooled. Ammonium sulfate was added slowly up to a saturation of 15 % and stirred on ice for 1 h. After filtration by a 0.45-μm cellulose acetate membrane, the solution was loaded in 1 ml/min onto a HiTrap Phenyl HP column (1-ml column). The linear gradient was applied after 20 min washing with sodium phosphate buffer (pH 7.0) and 2 M NaCl. After 30 min, the washing was stopped with 100 % sodium phosphate buffer (pH 7.0).

#### Protein digestion

An aliquot of the fraction was treated with a finale concentration of 10 mM dithiothreitol for 15 min in a water bath at 60 °C. Free cysteine residues were then alkylated with a finale concentration of 60 mM iodoacetamide in the dark for 15 min at room temperature. Reduced and alkylated fractions were either loaded on the SDS gel or directly digested by trypsin (1.6 μg/ml) overnight at 37 °C. In-gel digestion was preformed with the protocol from OMX system (Proteome X Solution, Germany).

#### HPLC analysis of digested proteins

The HPLC system (Ultimate 3000RS Dionex, Germany) used consisted of a degasser (SRD 3400), a pump module (HPG 3400RS), an auto sampler (WPS 3000TRS), a column compartment (TCC 3000RS), a diode array detector (DAD 3000RS), and an ESI-ion-trap (HCT Bruker, Germany). Data were collected and analyzed with Bruker HyStar and Data Analysis software. The Gravity column (100 mm length, 2 mm i.d., and 1.8 μm particle size, Macherey-Nagel, Germany) was tempered at 40 °C. Flow rate was set to 0.2 ml/min and the gradient was programmed as follows: mobile phase B started at 10 % for 5 min, increasing to 75 % over 45 min, and returning in 0.2 min to starting conditions for 4.8 min (mobile phase A, 0.1 % formic acid in water; mobile phase B, 0.1 % formic acid in acetonitrile). Temperature of the auto sampler was 10 °C and injection volume was set to 10 μl.

#### ESI-ion trap parameter

The ion trap was operated in the ultra standard enhanced mode (8,100 *m*/*z*/s) from *m*/*z* 300 to 1,500 (*m*/*z* 100 to 2,300 for MS/MS). The ICC target was set to 200,000 with a maximum accumulation time of 150 ms and five averages (three for MS/MS). The ion source parameters were set as follows: capillary voltage 4 kV, dry temperature 365 °C, nebulizer pressure 40 psi, and a dry gas flow of 9 l/min Auto MS mode with a smart target mass of 800 *m*/*z* and a MS/MS fragmentation amplitude of 0.5 was used.

### Enzyme assay

The ADH activity was determined photometrically by monitoring the increase/decrease of NADP^+^/NADPH at 340 nm in a Mulitskan® spectrum spectrophotometer (Thermo Fisher Scientific). The reaction mixture contained 50 mM Tris-HCl, pH 7.5, 0.3 mM NAD(P)H a defined aldehyde as substrate, and the purified enzyme at 37 °C. One unit of enzyme activity was defined as the amount of protein that oxidizes 1 μmol of NAD(P)H/min at 37 °C. Calculation of Michaelis–Menten kinetics for determination of *K*_m_ and *V*_max_ was done with SigmaPlot 11.0 (Systat Software). Alcohols with alkyl chains longer than C6 were not tested as substrates due to solubility problems.

### Enzyme expression and purification

Protein expression is exemplarily described for one enzyme and was performed for other proteins by the same procedure. *E*. *coli* BL21(DE3) containing the plasmid of interest was grown in 50 ml autoinduction media for efficient protein expression (Studier 2005). In the case of YjgB, YahK, and YqhD, additional ZnCl_2_ was added to the media with a final concentration of 0.1 mM. The preculture was incubated in 4 ml of LB medium with 100 μg/ml kanamycin at 37 °C overnight on a rotary shaker (180 rpm). Expression culture was inoculated with a 1:100 dilution of overnight culture. Incubation was performed foremost 3 h at 37 °C followed by incubation for 21 h at 16 °C. Cells were harvested by centrifugation and resuspended in 50 mM sodium phosphate buffer (pH 8.0, 20 mM imidazol, 500 mM NaCl, and 10 % glycerol). Crude extracts were prepared by use of a cell disrupter (IUL Instruments) and subsequent addition of MgCl_2_ to a final concentration of 2.5 mM in combination with DNase (1 μg/ml) and a following incubation for 20 min at room temperature for DNA degradation. The insoluble fraction of the lysate was removed by centrifugation at 20,000 rpm for 40 min at 4 °C. The supernatant was filtered through a 0.45-μm syringe filter and applied to an affinity resin column, 5 ml HisTrap^TM^ FF, equilibrated with the resuspension buffer using the ÄKTA UPC-900 FPLC-system. The enzyme was washed with 20 ml of resuspension buffer and eluted with 50 mM sodium phosphate buffer (pH 8.0, 500 mM imidazol, 500 mM NaCl, and 10 % glycerol). Aliquots of each eluted fraction were subjected to 12 % SDS-PAGE. The fractions containing the eluted protein were pooled and the protein was desalted using a HiPrep^TM^ 26/10 Desalting column which preliminary equilibrated with 50 mM Tris-HCl, pH 8.0. Protein concentrations were determined using a Bradford assay Roti®-nanoquant (Carl Roth).

### Determination of kinetic parameters

For the characterization of recombinant YahK and YjgB, enzyme activity was assayed for the reduction of carbonyl compounds and the oxidation of alcohols. For the reduction of carbonyl compounds, the assays were conducted at pH 7.5, 50 mM Tris-HCl, and 37 °C. The oxidation of alcohols was assayed with a slightly higher pH of 8.5, 50 mM Tris-HCl, and 37 °C. For every substrate, *K*_m_ and *k*_cat_ values were determined as well as for the corresponding cofactor NAD(P)^+^/NAD(P)H. Additionally, the activity of YqhD, YahK, and YjgB was determined at different temperatures using 50 mM Tris-HCl, pH 7.5, and 0.3 mM NADPH with butyraldehyde as the substrate. The decrease of NADPH at 340 nm was determined using a Shimadzu UV-1800 UV-spectrophotometer (Duisburg, Germany)

### Structure modeling

The structure of YjgB was modeled with “One to one threading” using YahK as template with Phyre2 (Kelley and Sternberg 2009). YahK was chosen as starting point due to the highest similarity (32 %) in a previous global modeling approach of Phyre2. In the crystal structure of YahK, the section between amino acid 269 and 277 was not resolved. As this part does not seem to be involved in the catalytic mechanism, it was neglected. Using 3DLigandSite, the cofactor NADPH was integrated into the structure for both proteins (Wass et al. 2010). Finally the substrate butyraldehyde was docked into the active site using YASARA (www.yasara.org) and energy minimization was performed using the force field AMBER99.

## Results

### Analysis of reductive activity of *E*. *coli*

Since 1997, the complete genome of *E*. *coli* is accessible and mostly annotated; however, nearly 10 % of its genes with their potentially encoded proteins are still unidentified (Blattner et al. 1997; Riley et al. 2006; Feist et al. 2007). In addition, the exact activity of many of the annotated genes is not known. We were interested to find which enzymes in *E*. *coli* are responsible for the major NADPH-dependent aldehyde reductase activity. Within the genome of *E*. *coli*, more than 20 enzymes can be identified that could be potentially important for this reaction.

**Fig. 1:**
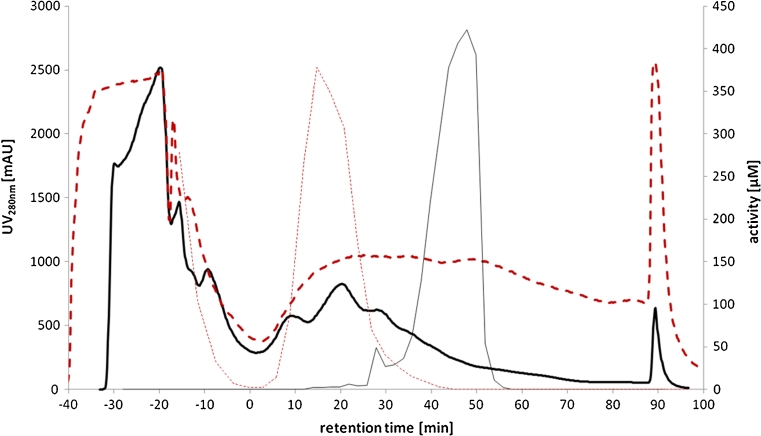
AXC purification with normalized RT because of different loading volumes (gradient starts at 0 min), negative retention time shows loading and wash out unbound sample: UV_280nm_ signal from purification of *E*. *coli* wild type (*thick solid line*), analyzed fraction (1.5 ml) activity of *E*. *coli* wild type (*thin solid line*), UV_280nm_ signal from purification of *E*. *coli* Δ*yahk* (*thick broken line*), analyzed fraction (1.5 ml) activity of *E*. *coli* Δ*yahk* (*thin broken line*)

**Fig. 2:**
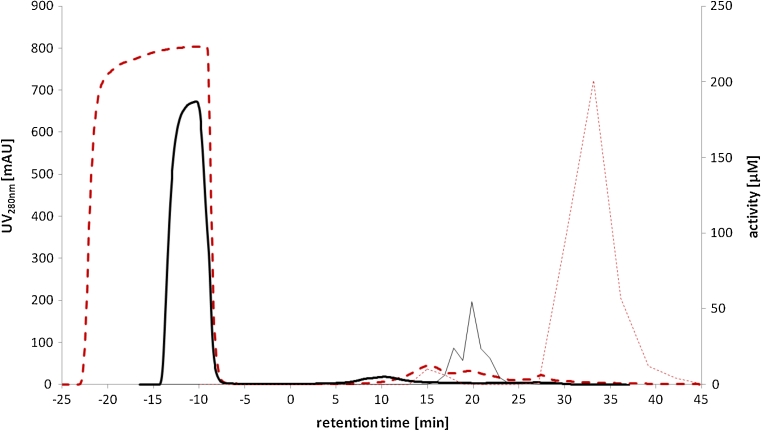
HIC purification with normalized RT because of different loading volumes (gradient starts at 0 min), negative retention time shows loading and wash out unbound sample: UV_280nm_ signal from purification of *E*. *coli* wild type (*thick solid line*), analyzed fraction (1.5 ml) activity of *E*. *coli* wild type (*thin solid line*), UV_280nm_ signal from purification of *E*. *coli* Δ*yahk* (*thick broken line*), analyzed fraction (1.5 ml) activity of *E*. *coli* Δ*yahk* (*thin broken line*)

**Fig. 3:**
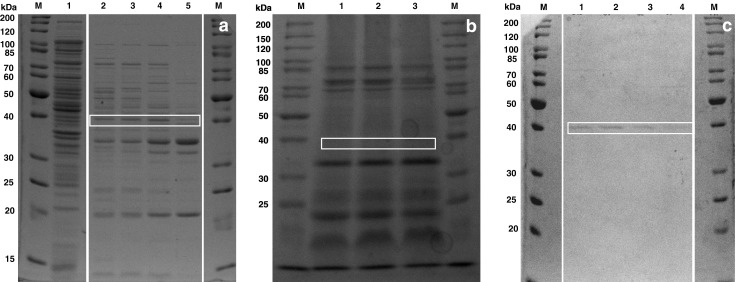
SDS gel (12 %) with *M* PageRuler unstained protein ladder (Fermentas): **a** fractions of AXC purification from *E*. *coli* wild type, *1* crude extract; *2*–*5* 1 ml active fraction from RT 45.8–49.8 min; **b** AXC from *E*. *coli* Δ*yahk*, *1*–*3* fractions without YahK band (no activity) from RT 46.8–49.8 min; **c** fraction of HIC purification from *E*. *coli* wild type, *1*–*4* 1 ml active fraction from RT 26.8–30.8 min

We therefore purified respective enzymes from cell lysate and identified them by protein sequencing. In short, lysed cells of an *E*. *coli* culture grown on LB medium were prepared and enzymes were partially purified by anion exchange chromatography. All eluted fractions were analyzed for their NADPH-dependent aldehyde reductase activity. One major activity peak was detected (Fig. 1). All active fractions were collected and subjected to a hydrophobic interaction chromatography (Fig. 2). Analysis of the active fractions on an SDS-PAGE showed one single band corresponding to the NADPH-dependent reductase activity (Fig. 3a, c). BLAST analysis of sequencing results of the protein band from the SDS gel by MS revealed it to be YahK (37,954 Da), a so far uncharacterized oxidoreductase.

To confirm this result, a deletion of *yah*K in *E*. *coli* was examined. We therefore analyzed Δ*yah*K strain (JW0317) from Keio collection, in the same way as wild type of *E*. *coli* W3110. Anion exchange chromatography of the knockout variant showed a new enzyme activity eluting earlier as the YahK activity of the wild type *E*. *coli* (Fig. 1). No corresponding band to YahK (∼38 kDa) could be detected by the SDS gel (Fig. 3b).

In the hydrophobic interaction chromatography (HIC) purification (Fig. 2), the newly identified activity showed longer retention time (RT) than YahK with a low UV signal at 280 nm. By SDS gel electrophoresis no detectable band was identified (data not shown). For that reason, the complete active fraction was subjected to a tryptic digestion and analyzed via MS. Sequencing of the fraction revealed the enzyme to be YjgB, another so far uncharacterized zinc-type alcohol dehydrogenase-like protein.

### Homologue expression

The genes of YjgB and YahK as well as of the previously characterized YqhD were amplified by PCR, cloned, and expressed in *E*. *coli* BL21 (DE3). Comparison between the cell pellet and the cell-free soluble extracts revealed the formation of inclusion bodies. The ratio of insoluble and soluble enzyme was 50:50 (data not shown). The enzymes were purified via an N-terminal His tag for the determination of the kinetic parameters. The purified proteins appeared as single band on SDS polyacrylamide gels. The molecular weight was calculated to be 40.43 kDa for YahK and 38.66 kDa for YjgB (including the additional amino acids of the His tag). After 14 days at 8 °C in desalting buffer (50 mM Tris-HCl pH 8.0), YahK, YjgB, and YqhD still exhibited 85 % of the initial activity. For long-term storage at −20 °C, glycerol (25 % *v*/*v*) was added and no loss of activity was observed after 4 months for all three enzymes.

### Substrate and cofactor specificity of YahK and YjgB

The purified enzymes were used to determine their kinetic parameters *k*_cat_ and *K*_m_ for different substrates. Various aldehydes and alcohols were used as substrates, and also the NAD(P)^+^/NAD(P)H cofactor concentration was varied to determine the corresponding *K*_m_ (Table 1). The *K*_m_ values towards the different substrates varied between 0.22 and 193.7 mM for YjgB and 0.135 and 52.6 mM for YahK. Both enzymes strongly prefer aldehyde compounds and no activity was measured using a ketone as substrate. NADPH cannot be substituted by NADH for both enzymes without losing >99 % of the activity. For both enzymes, hexanal represents the best substrate concerning the turnover number.

**Table 1:** Kinetic parameters determined for YjgB, YahK, and YqhD* corresponds to data from Jarboe (2010) for different substrates

Enzyme	Cofactor	Substrate	*K*_m_ (mM)	*k*_cat_ (s^−1^)	*k*_cat_/*K*_m_ (s^−1^ mM^−1^)
YjgB	NADPH	Acetaldehyde	73.4 ± 5.4	332.6 ± 26.1	4.5
	NADPH	Propionaldehyde	19.6 ± 0.8	207.2 ± 2.9	10.6
	NADPH	Glyceraldehyde	n.c.a.	n.c.a.	n.c.a.
	NADPH	Butyraldehyde	2.1 ± 0.09	464.4 ± 24.2	221
	NADPH	Isobutyraldehyde	193.7 ± 27.5	50.8 ± 9.4	0.26
	NADPH	Crotonaldehyde	2.4 ± 0.2	77.6 ± 3.3	32.3
	NADPH	2-Butanone	n.c.a.	n.c.a.	n.c.a.
	NADPH	Glutaraldehyde	5.8 ± 0.5	311.9 ± 14.8	53.8
	NADPH	5-Hydroxyvalerate	59.7 ± 3.5	2.6 ± 0.04	0.04
	NADPH	Hexanaldehyde	0.34 ± 0.03	418.9 ± 15.7	1,232
	NADPH	Benzaldehyde	0.24 ± 0.02	313.0 ± 7.4	1,305
	NADPH	Furfural	0.22 ± 0.02	224.13 ± 26.9	1,018
	NADP^+^	Butanol	3.5 ± 0.28	13.79 ± 0.6	3.94
	NADP^+^	1,4-Butanediol	24.1 ± 1.5	12.9 ± 0.4	0.53
	Butanol	NADP^+^	0.076 ± 0.01	14.37 ± 0.2	189
	Butyraldehyde	NADPH	0.06 ± 0.003	224.1 ± 36.5	3,734
YahK	NADPH	Acetaldehyde	13.3 ± 1.0	11.18 ± 0.7	0.84
	NADPH	Propionaldehyde	10.9 ± 1.0	11.6 ± 0.6	1.1
	NADPH	Glyceraldehyde	4.4 ± 0.4	12.3 ± 1.3	2.8
	NADPH	Butyraldehyde	2.1 ± 0.1	41.6 ± 0.6	19.8
	NADPH	Isobutyraldehyde	2.2 ± 0.2	32.1 ± 1.2	14.6
	NADPH	Crotonaldehyde	3.6 ± 0.4	32.6 ± 2.1	9.1
	NADPH	2-Butanone	n.c.a.	n.c.a.	n.c.a.
	NADPH	Glutaraldehyde	4.1 ± 0.4	13.4 ± 1.5	3.3
	NADPH	5-Hydroxyvalerat	52.6 ± 13.2	0.18 ± 0.02	0.003
	NADPH	Hexanaldehyde	0.37 ± 0.02	18.3 ± 0.6	49.5
	NADPH	Benzaldehyde	0.29 ± 0.02	7.75 ± 0.5	26.7
	NADPH	Furfural	0.135 ± 0.03	12.5 ± 1.9	92.3
	NADP^+^	Butanol	6.6 ± 0.3	4.7 ± 0.07	0.71
	NADP^+^	1,4-Butanediol	38.5 ± 2.9	6.7 ± 0.3	0.18
	Butanol	NADP^+^	0.012 ± 0.001	7.47 ± 0.07	622.0
	Butyraldehyde	NADPH	0.011 ± 0.001	9.8 ± 0.4	894.4
YqhD*	NADPH	Acetaldehyde	30	1.1	0.033
	NADPH	Propanaldehyde	3.3	45	14
	NADPH	Butyraldehyde	0.67	60	87
	NADPH	Isobutyraldehyde	2	1	0.5
	NADPH	Glyceraldehyde	1.4	3.4	2.4
	NADPH	Furfural	9	n/a	n/a
	NADPH		0.008	n/a	n/a

*n*.*c*.*a*. no catalytic activity, *n*/*a* not available

YahK shows lower *K*_m_ values than YjgB for linear aliphatic aldehydes (factor of 10 for acetaldehyde, 2 for propionaldehyde, or 1.5 for hexanal). This difference is much more pronounced for the branched aldehyde isobutanal. Here the *K*_m_ of YjgB is 64 times higher. For tested substrates carrying additional functional groups and such being more polar are mostly better recognized by YjgB. Crotonaldehyde and 1,4-butanediol have lower *K*_m_ values with YjgB, whereas glutaraldehyde and 5-hydroxyvalerate have almost the same *K*_m_ values with YjgB and YahK. Glyceraldehyde shows an intermediate behavior, which is reasonable as it is carrying additional functions but can be considered branched as well and is only accepted by YahK as substrate. Recognition of aromatic aldehydes is similar for both enzymes. Turnover numbers show a more uniform behavior; generally they are higher with YjgB than YahK. This is more pronounced with aliphatic aldehydes as substrates (factors between 5 and 20 between the two enzymes) than for bi- and trifunctional molecules (factors between 3 and 10). Aromatic molecules are converted faster with YjgB as well. In summary, YjgB has the higher catalytic efficiency for most substrates, isobutyraldehyde being the one exception. YahK and YjgB show inhibition by some substrates (hexanal, benzaldehyde, and furfural at concentrations higher than ca. 2 mM). We compared YjgB and YahK with kinetic data published for YqhD (Table 1). This has to be considered with caution, as test conditions for YqhD varied. But in general, YqhD has the lower *K*_m_ values but also much lower turnover numbers leading to an overall reduced catalytic efficiency.

**Fig. 4:**
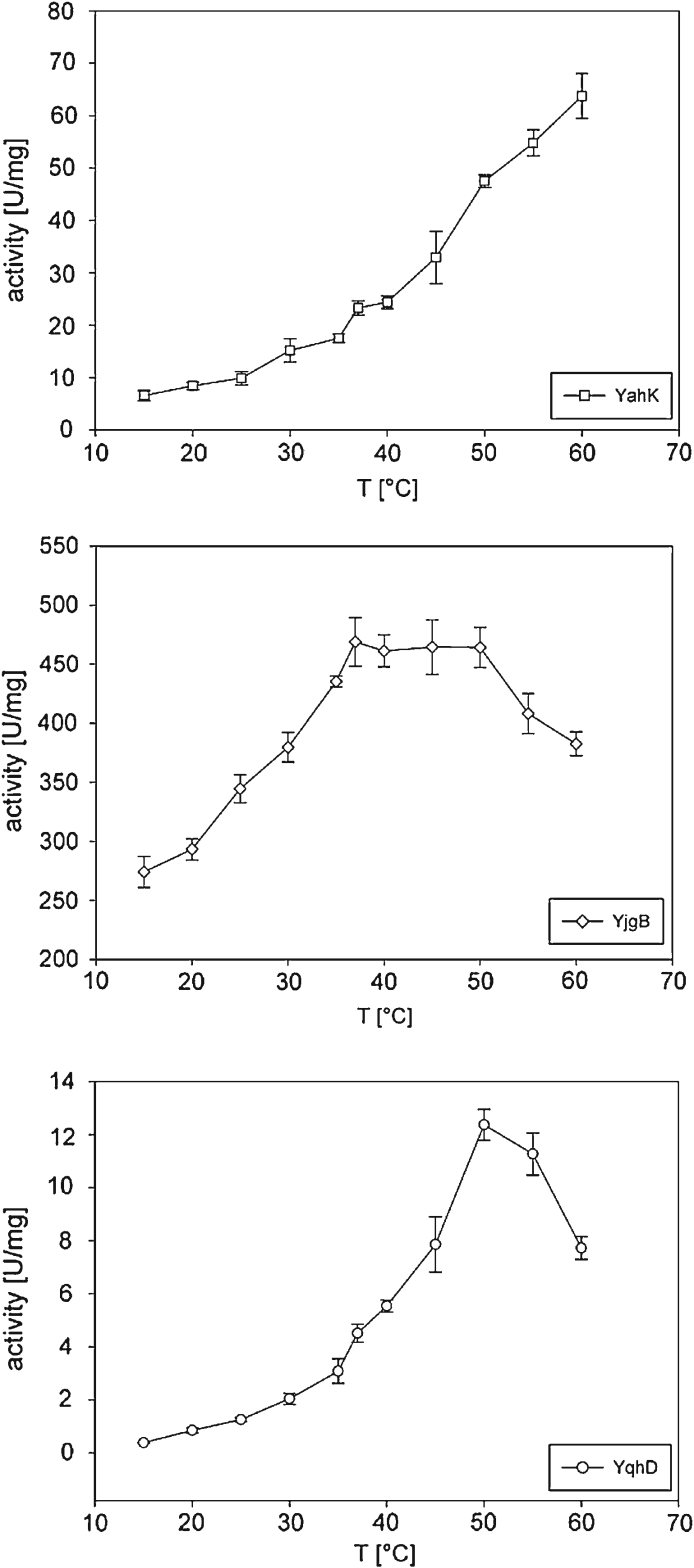
Specific activity of Yahk, YjgB, and YqhD at different temperatures; values were determined by photometric assay in 50 mM Tris-HCl buffer at pH 7.5, 5 mM butyraldehyde, and 0.3 mM NADPH for different temperatures

Activity of YqhD, YahK, and YjgB was determined in the temperature range from 15 to 60 °C using butyraldehyde as substrate (Fig. 4). Every enzyme showed a unique temperature profile. YahK appears to be stable up to 60 °C; there is a constant increase in activity up to 65 U/mg at 60 °C. Interestingly, the specific activity of YjgB remained constant at around 460 U/mg between 37 and 50 °C, above which it declined. YqhD showed an increase in activity until 50 °C with 12 U/mg. At higher temperatures the activity decreases.

### Sequence and structural comparison

**Fig. 5:**
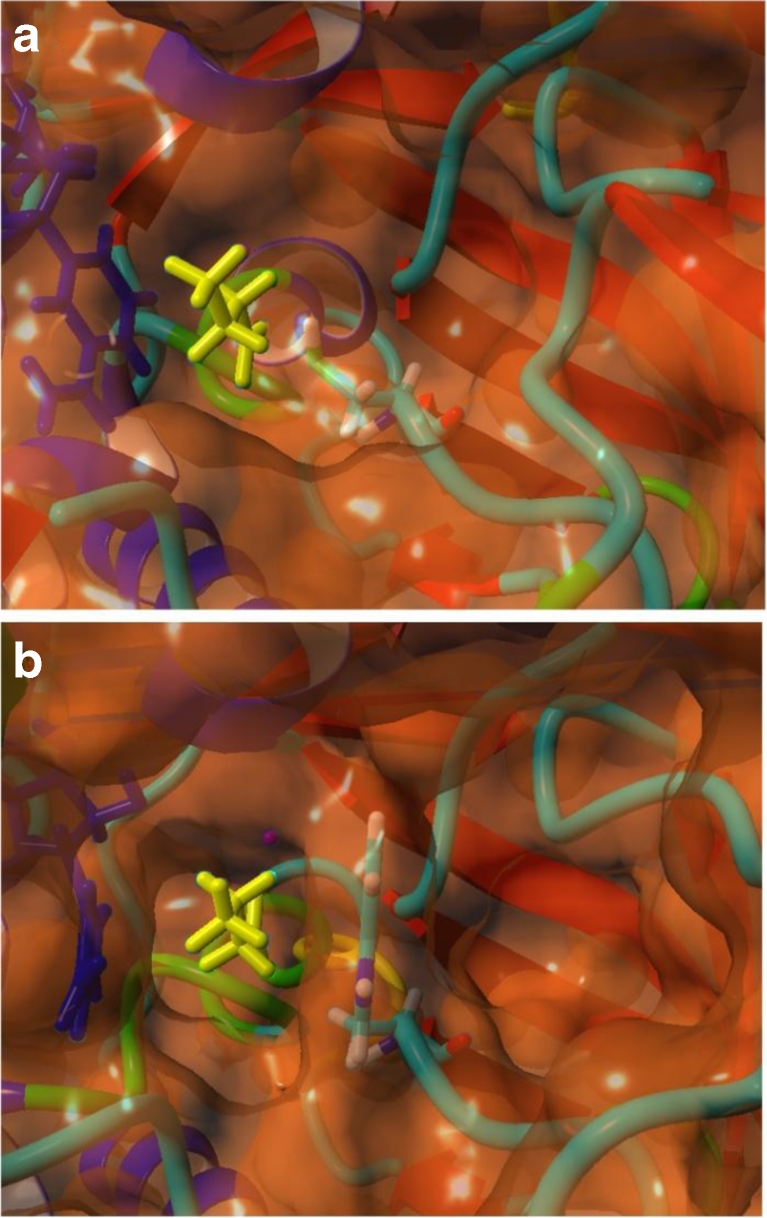
The Substrate Binding Pocket for **a** YahK (PDB 1UUF) and **b** YjgB. Docking solutions for butyraldehyde (*yellow*) and the cofactor NADPH (*blue*), respectively. The substrate is targeted through interaction of the aldehyde function with the Zn^2+^ ion (*purple*)

The size of YahK is 349 amino acids and for YjgB 339 amino acids in accordance to Jörnvall et al. (1999a), both are classified as medium-chain dehydrogenases (Nordling et al. 2002). An alignment for all 17 possible medium-chain dehydrogenases/reductases (MDR) enzymes of *E*. *coli* revealed the closest relationship of YahK and YjgB to each other without mentioning YqhD (Jörnvall et al. 1999b). All three enzymes are grouped to the MDR superfamily, YahK and YjgB belong to the cinnamyl alcohol dehydrogenase family, whereas YqhD to the polyol dehydrogenase family (Cambillau et al. 2004; Persson et al. 2008). YahK as well as YjgB possess the GHEX_2_GX_5_(G,A)*X*_2_(I,V,A,C,S) protein pattern that can be found in Zn-containing MDRs and the GX_1-3_GX_1-3_G pattern located in the nucleotide-binding region. There exists an entry in the protein data bank (PDB entry 1UUF) for YahK without any further information. The structure was solved like that of YqhD in a structural genomics program determining the crystal structures of *E*. *coli* open reading frame (ORF) products of unknown function (Sulzenbacher et al. 2002; Vincentelli et al. 2003). We modeled the structure of YjgB based on the YahK structure to compare the active sites of both enzymes (Fig. 5). From this perspective in both cases, the substrate is embedded through the cofactor on one side and coordinated through the Zn^2+^ ion. There is a pronounced difference between both enzymes in the active center. YahK exhibits more space through a cysteine at position 88, whereas in YjgB, the corresponding residue is a tryptophane (AS 91) that limits the available space. This substantially reduces the size of the substrate binding pocket. Such in YjgB, the space between the cofactor and the catalytic zinc represents a short hollow tube. The differences in shape of the substrate binding pocket could explain the different substrate preference of the two enzymes. As shown above, YjgB strongly prefers unbranched substrates, which is well demonstrated by the marked difference between *n*-butyraldehyde and isobutyraldehyde. In contrast, both substrates react very similar in YahK. When tryptophane 91 was removed, the cavity in YigB would be similar to that of YahK or even slightly larger.

## Discussion

In analyzing enzymes of *E*. *coli* responsible for reducing aldehydes to primary alcohols, we found two so far uncharacterized enzymes YahK and YjgB to be relevant. Interestingly, we could not detect YqhD, an enzyme which earlier has been reported to be important (Atsumi et al. 2009; Jarboe 2010; Nakamura and Whited 2003). YjgB appears to be the more active enzyme; however, its production seems to be only induced when the gene coding for YahK is knocked out. Further studies on conditions that lead to the induction of YjgB in comparison to YqhD and YahK have to be performed. The in vivo functions of YahK as well as YjgB have yet to be determined. Due to their similar substrate specificities compared to YqhD, a related function is possible. First reports of the in vivo function of YqhD describe involvement in a NADPH-dependent response mechanism to lipid peroxidation (Perez et al. 2008). Additionally, expression analysis concerning growth-limiting conditions using *E*. *coli* K-12 W3110 and comparison between wild type and cold-sensitive deletion strains revealed an upregulation of YqhD (Phadtare and Inouye 2004; Hua et al. 2004). Until now, no study dealing with the investigation of global responses of *E*. *coli* focusing on an altered expression profile identified *yah*K and *yjg*B and could connect it with a special enzyme activity. Cinnamyl alcohol dehydrogenases catalyze the last step in the biosynthesis of monolignols in plants (Sibout et al. 2005), which is hardly relevant in *E*. *coli*. Enzymes of this class have been found important in NADP/NADPH homeostasis, lipid biosynthesis, amino acid metabolism, or the formation of fusel alcohols and have been discussed for enzymes of this family (Larroy et al. 2002, 2003). The rather broad substrate range of YahK and YjgB implies a more universal function like the mentioned NADP/NADPH homeostasis. This is an interesting fact in combination to ongoing research regarding the use of lignocellulosic hydrolysates as a cheap carbon source for fermentations. Depending on the type of biomass, pretreatment hydrolysates can contain significant amounts of furfural (app. 1 g l^−1^) which has a tremendous effect on growth of *E*. *coli* (Miller et al. 2009a; Almeida et al. 2009). Different strategies for use of hydrolysates are investigated whereas adapted strains represent the most elegant way. A first attempt in an adaptation process generated an *E*. *coli* mutant EMFR9 with downregulated expression of *yqh*D (Miller et al. 2010). The efficient detoxification of furfural leads to an imbalance in the NADPH pool. This results from the low *K*_m_ of YqhD for NADPH (8 μM) (Miller et al. 2009b). This extreme low *K*_m_ stands in contrast to the eightfold higher *K*_m_ of YjgB. The newly identified dehydrogenases can be an interesting starting point for further strain improvement in this way.

*E*. *coli* K-12 is supposed to contain 17 ORFs that are encoding MDR alcohol dehydrogenase but without identification of YqhD as a MDR (Jörnvall et al. 1999a, b). So apparently, the well examined workhorse *E*. *coli* still bears some biocatalysts which are promising candidates for metabolic engineering approaches for the optimized production of bulk chemicals and that might be better suited than currently applied enzymes. In this context, we suggest to rename all characterized Zn-dependent- and NADPH preferring alcohol dehydrogenases from *E*. *coli* YqhD, YahK, and YjgB to AdhZ1, AdhZ2, and AdhZ3, respectively. The newly characterized enzymes AdhZ2 and AdhZ3 should be considered, when knockout variants are prepared that are not supposed to reduce aldehydes, as for example shown in the production of isobutyric acid (Zhang et al. 2011). AdhZ3 seems to be especially useful for the conversion of bifunctional substrates and AdhZ2 for branched chain substrates. So far, the focus in metabolic engineering has been on AdhZ1 as it also accepts a wide range of carbonyl compounds rendering it an interesting candidate for various mutation approaches though at lower activity (Jarboe 2010; Tang et al. 2009; Liao et al. 2010). First engineering approaches for a further improvement of the enzyme resulted in variants with increased catalytic efficiency on 3-hydroxypropionaldehyde (Li et al. 2008). Using the more active AdhZ3 or AdhZ2 instead might be a better alternative.
